# Gender difference in N170 elicited under oddball task

**DOI:** 10.1186/s40101-015-0045-7

**Published:** 2015-03-04

**Authors:** Damee Choi, Yuka Egashira, Jun’ya Takakura, Midori Motoi, Takayuki Nishimura, Shigeki Watanuki

**Affiliations:** Department of Kansei Science, Kyushu University, 4-9-1 Shiobaru, Minami-ku, Fukuoka, 815-8540 Japan; Department of Public Health, Nagasaki University Graduate School of Biomedical Sciences, 1-12-4 Sakamoto, Nagasaki, Japan; Faculty of Design, Kyushu University, 4-9-1 Shiobaru, Minami-ku, Fukuoka, 815-8540 Japan

**Keywords:** Gender difference, N170, Event-related potential, Attention, Face, Oddball task

## Abstract

**Background:**

Some studies have reported gender differences in N170, a face-selective event-related potential (ERP) component. This study investigated gender differences in N170 elicited under oddball paradigm in order to clarify the effect of task demand on gender differences in early facial processing.

**Findings:**

Twelve males and 10 females discriminated targets (emotional faces) from non-targets (emotionally neutral faces) under an oddball paradigm, pressing a button as quickly as possible in response to the target. Clear N170 was elicited in response to target and non-target stimuli in both males and females. However, females showed more negative amplitude of N170 in response to target compared with non-target, while males did not show different N170 responses between target and non-target.

**Conclusions:**

The present results suggest that females have a characteristic of allocating attention at an early stage when responding to faces actively (target) compared to viewing faces passively (non-target). This supports previous findings suggesting that task demand is an important factor in gender differences in N170.

## Background

Many psychological [1-3] and physiological [4-16] studies have revealed gender differences in facial processing. Wood and Eagly [17] argued that gender differences in behaviours might be generated from biological specialization, such as male physical attributes (size, strength and speed) and female reproductive capacity. From an anthropological perspective, gender differences in face processing are thus thought to be related to the differing social roles of males and females.

Some event-related potential (ERP) studies [4-9] have also investigated gender differences in facial processing, using N170 as the index of attention. N170 is an ERP component showing a negative peak around 170 ms after face onset in the posterior temporal area [18-22] and is thus considered a face-selective ERP component. Given that N170 is more negative when faces are attended than when faces are presented outside the attentional focus [21], a more negative N170 appears to reflect increased attention to faces. Sun et al. [4] revealed that females showed a more negative amplitude of N170 when discriminating orientations (right or left) of faces than genders of faces, while males did not. From this result, the authors suggested that the effect of task demands on N170 is more obvious in females than in males [4]. However, to the best of our knowledge, very few studies have investigated the effect of task demand on gender differences in N170 [4,9]. This issue therefore remains unclear.

To clarify how task demand affects gender differences in N170, examining whether males and females show differences in N170 between reacting to faces actively (for example, pressing a button) and viewing faces passively seems to be appropriate. The present study thus aimed to investigate gender differences in N170 elicited under an oddball task by reanalyzing data from our previous study [23]. The oddball task is a well-studied paradigm in which two types of stimuli are presented and the participant is usually instructed to press a button in response to one type of stimulus (the target). In the present study, target stimuli were emotional faces (happy, angry, surprised, afraid or sad), while non-target stimuli were emotionally neutral faces. We hypothesized that females, compared to males, would show greater difference in N170 when responding to target and non-target faces, given that females show greater N170 modulation by task demand [4].

## Methods

### Participants

Twenty-two healthy, right-handed undergraduate and graduate students (12 males: age range, 21 to 25 years; 10 females: age range, 22 to 28 years) participated in this study. All participants provided written informed consent. The study was approved by the ethics committee in the Department of Design at Kyushu University, Japan.

### Stimuli and procedures

We selected images of 12 adult humans (six men, six women, 20 to 30 years of age, Caucasian) showing six types of facial expression (neutral, happy, angry, surprised, afraid or sad) from Karolinska Directed Emotional Faces [24]. All images were edited to square of 300 × 400 pixels and presented in the centre of a black screen (17-inch monitor, 1,024 × 768 resolution). The distance between the participants and the monitor was 70 cm, and the images subtended approximately 6° × 6° of visual angle.

Electroencephalography (EEG) was recorded during five blocks of oddball tasks. Each block consisted of 96 trials. Non-target stimuli (presented in 75% of trials) were emotionally neutral faces in all blocks, whereas target stimuli (presented in 25% of trials) were happy, angry, surprised, afraid or sad faces in each block. Participants responded to target stimuli by pressing a button using the right hand. In each trial, a cross shape was presented (500 ms), followed by a target or non-target image (800 ms). The interstimulus interval was 1,000 ms, and targets were never presented consecutively.

### ERP measurements and analysis

We recorded EEG with averaged ears as the reference using a Polymate AP1532 system (TEAC, Tokyo, Japan) from the following five sites: Fz (medial frontal), Cz (medial central), Pz (medial parietal), T5 (left posterior temporal) and T6 (right posterior temporal). We also recorded electrooculography (EOG) to detect blinking with electrodes above and below the right eye. The impedance of each electrode was kept below 10 kΩ.

EEG signals were digitized at a sampling rate of 500 Hz, and a band-pass filter of 1 to 30 Hz was applied (EMSE Suite; Source Signal Imaging, San Diego, CA, USA). Target and non-target stimulus presentation of −200 to 800 ms was averaged across blocks (baseline: −200 to 0 ms). Trials containing artefacts >50 μV and trials in which the subject did not respond were excluded from averages. For target stimuli, the mean number of trials was 106.4 (standard deviation (SD) = 8.8) and 97.1 (SD = 8.8) in males and females, respectively. For non-target stimuli, the mean number of trials was 322.3 (SD = 29.6) and 288.6 (SD = 54.5) in males and females, respectively.

We calculated N170 as the most negative potential within 140 to 200 ms at the T5 and T6 sites, where N170 amplitude has been reported to be most negative [18-22] and to show gender differences in asymmetry [5-8].

### Statistical analysis

For ERP responses, we conducted repeated-measures analysis of variance (ANOVA) with gender as a between-subject factor and task (target and non-target) and site (T5 and T6) as within-subject factors. For behavioural data (response accuracies and reaction times), the independent *t*-test was used for comparisons between males and females.

Statistical significance was accepted at the 5% level (*P* < 0.05) (SPSS, Chicago, IL, USA). The Greenhouse-Geisser correction was applied where sphericity was violated. When the main effect or an interaction was significant, pairwise comparisons were performed with the Bonferroni correction.

## Results

**Figure 1:**
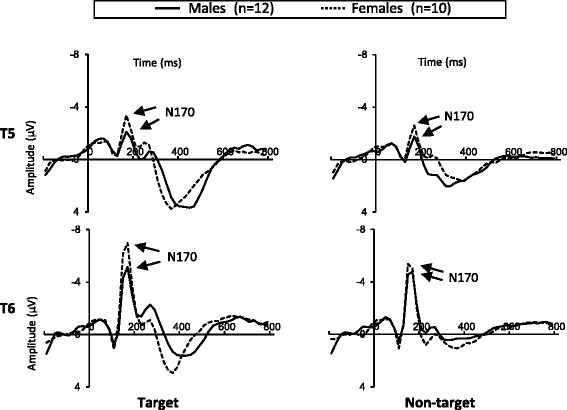
**Grand-averaged event-related potential (ERP) waveforms elicited by target (right column) and non-target (left column) at T5 and T6 sites.** Solid line indicates male participants (*n* = 12), and dotted line indicates female participants (*n* = 10).

**Figure 2:**
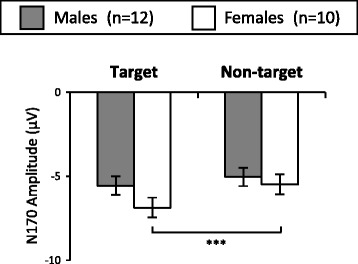
**N170 amplitude elicited by target and non-target (mean and standard deviation).** Grey bar indicates male participants (*n* = 12) and white bar indicates female participants (*n* = 10). ***P < 0.001 (pairwise comparison between N170 elicited by target and non-target in females).

Figure 1 shows ERP waveforms elicited at T5 and T6 sites. For N170, a reliable interaction of gender and task (*F*(1,20) = 4.62, *P* < 0.05) was seen, suggesting that N170 was more negative in response to target than in response to non-target in females (*P* < 0.001) but not in males (*P* > 0.05) (Figure 2). The main effect of task was significant (*F*(1,20) = 22.65, *P* < 0.001), due to the fact that N170 was more negative in response to target than in response to non-target (Figure 1). The main effect of site was also significant (*F*(1,20) = 11.98, *P* < 0.001), indicating that N170 was more negative at T6 than at T5 (Figure 1). No significant effects of gender or other interactions were seen (*P* > 0.05).

**Figure 3:**
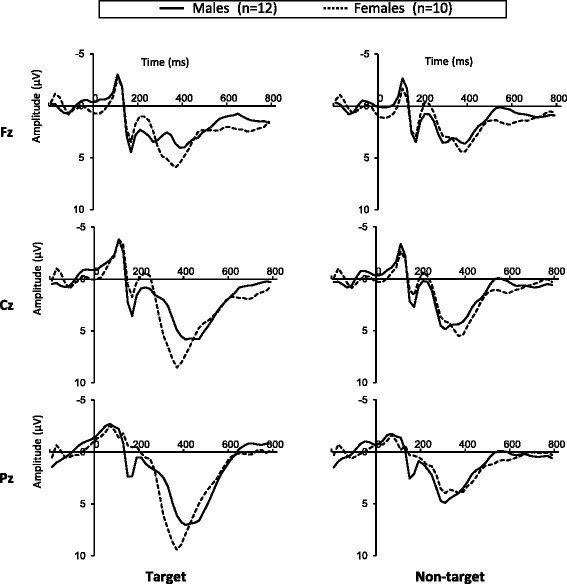
**Grand-averaged event-related potential (ERP) waveforms elicited by target (right column) and non-target (left column) at Fz, Cz and Pz sites.** Solid line indicates male participants (*n* = 12), and dotted line indicates female participants (*n* = 10).

Figure 3 shows ERP waveforms elicited at Fz, Cz and Pz sites for an understanding of the overall EEG pattern.

No significant gender differences in response accuracies or reaction times were seen (response accuracies: *t* = −1.85; reaction times: *t* = 0.81; all df = 20, all *P* > 0.05).

## Discussion

The present study investigated gender differences in N170 elicited under the oddball task, in order to identify effects of task demand on gender differences in early facial processing. We found that females showed more negative N170 in response to target than non-target, whereas males did not show any difference in N170 between response to target and non-target (Figure 2). This suggests that females tend to show increased early attention when responding to faces actively (target) compared to viewing faces passively (non-target). This finding supports a previous study [4] that suggested females, compared to males, are more sensitive to N170 modulation by task demand.

One possible explanation of the present result is biological specialization between males and females. As mentioned in the introduction, females generally have less physical attributes (size, strength and speed) compared to males and have traditionally played a social role in raising children through gauging emotional states of infants from their faces [17]. Thus, in terms of survival for themselves and their children, females might have needed to be especially sensitive to facial expressions requiring active response, compared to males.

On the other hand, the current results indicated that both males and females showed more negative N170 in the right posterior temporal area (T6) than in the left posterior temporal area (T5) (Figure 1). This is not consistent with previous studies that have reported gender differences in the hemispheric asymmetry of N170 [5-8]. In those studies [5,6], males showed right hemispheric dominance of N170, whereas females showed N170 over both right and left hemispheres. At this point, explaining this difference between the previous and present results relating to asymmetry in N170 seems to be difficult, and future research is thus needed to address this question.

Several limitations must be considered when discussing the present results. First, the number of participants (12 males and 10 females) was smaller than previous studies of gender difference in N170 (14 males and 14 females [4]; 20 males and 20 females [5]; 25 males and 25 females [6]). Second, target faces were emotional faces and non-target faces were emotionally neutral faces in the present study. The possibility must be considered that increased attention to targets in females might be affected by not only the effect of target but also the effect of emotion. Further research is warranted to clarify this issue.

In conclusion, we found more negative N170 elicited by target than non-target in females but not in males. This suggests that only females might show increased early-stage attention when actively responding to faces than when viewing faces passively. Task demand thus seems to be an important factor in gender differences in N170, as suggested by previous studies.

## Abbreviations

ANOVA: analysis of variance
EEG: electroencephalography
EOG: electrooculography
ERP: event-related potential
